# Stimulating Customer Inspiration Through Online Brand Community Climates: The Mediating Role of Customer Interaction

**DOI:** 10.3389/fpsyg.2021.706889

**Published:** 2021-08-17

**Authors:** Yao Cao, Zhimin Zhou, Salman Majeed

**Affiliations:** ^1^Department of Marketing, College of Management, Shenzhen University, Shenzhen, China; ^2^Faculty of Humanities, Curtin University, Perth, WA, Australia; ^3^International Center for Hospitality Research and Development, Dedman College of Hospitality, Florida State University, Tallahassee, FL, United States

**Keywords:** organizational climate, customer inspiration, customer interaction, brand marketing, consumer behavior, online brand communities

## Abstract

This study examines the impacts of the online brand community (OBC) climate on customer interaction and customer inspiration, which are yet under-explored in the extant literature. The data were collected online from the Chinese respondents (*N* = 504) to analyze the proposed constructs of the study. Findings show that supportive OBC climate and controlled OBC climate are positively related to customer interaction (including information interaction and social interaction) and exert a significant and positive impact on customer inspiration. A mediating impact of customer interaction is found on the relationship between OBC climate and customer inspiration. This study unravels the importance and mechanism of customer-brand relationships in the online environment and illuminates pathways for marketers and policymakers to positively influence customer inspiration for business promotion. This study updates existing literature boxes of consumer behavior and marketing in the context of online customer-brand relationships. Limitations and future research directions are noted.

## Introduction

Inspiration is a state that all humans experience, and for this reason, numerous studies in the fields of psychology, education, and management have been dedicated to inspiration (Wartiovaara et al., 2019; Khoi et al., 2020). Marketing scholars have also examined customer inspiration (Böttger et al., 2017; Winterich et al., 2019; Izogo and Mpinganjira, 2020) and noted that inspiration is an important component of customer experience (Liu et al., 2017). Customer inspiration refers to a temporary motivational state of customers which facilitates the transition from the reception of a marketing-induced idea to the intrinsic pursuit of a consumption-related goal (Böttger et al., 2017, p. 116). Some studies emphasize that customer inspiration is highly associated with brand attachment (Park et al., 2013), customer’s feeling of delight and satisfaction (Finn, 2005), and the flow of the customer experience (Schouten et al., 2007).

In marketing, the notion of customer inspiration has rarely been examined as a way to improve the management of customer relationship. Management of customer relationship is generally understood as a set of philosophies, strategies, systems, and technologies that assist brands and companies to manage their transactions and relationships with customers (Thakur and Workman, 2016). Previous studies on the management of customer relationship have mainly focused on analyzing how customer acquisition, retention, cross-selling, and loyalty programs impact the performance of companies (Ernst et al., 2011; Sigala, 2018; Dewnarain et al., 2019). However, the role of inspiration in the management of customer relationship is yet under-explored. While the management of customer relationship is widely accepted as an approach to develop and maintain long-term relationships with customers (Li et al., 2019), it is thus necessary to examine the potential of customer inspiration in management of customer relationship.

Meanwhile, with advances in information technologies, online brand communities (OBCs), in particular, the social-media-based OBCs, have become a vital platform for marketing. They are considered effective tools for initiating and reinforcing the relationship between a brand and its customers (Gómez-Suárez et al., 2017; Coelho et al., 2018). It is noted that OBCs help fuel brand love (Coelho et al., 2019) alongside fostering brand identification, commitment, and loyalty (Zhou et al., 2012; Brodie et al., 2013; Black and Veloutsou, 2017). However, the role of OBCs in stimulating customer inspiration is yet under-explored. Considering the growing body of literature on the role of augmented reality (AR) marketing and digital apps in promoting customer inspiration (Rauschnabel et al., 2019; Izogo et al., 2020), critical questions exist in understanding how to utilize OBCs to promote customer inspiration.

Online brand communities are of growing importance in the branding and marketing literature (Muniz and O’Guinn, 2001; Wirtz et al., 2013; Coelho et al., 2018). Recent research on brand community predominantly focused on investigating brand tribalism (Özbölük and Dursun, 2017), customer engagement (Hook et al., 2018; Yoshida et al., 2018; Matute et al., 2019), and value co-creation (Sanz-Blas et al., 2019; Chang et al., 2020). However, existing studies are yet to examine OBCs from the perspective of customer inspiration.

In addition, customer interaction is an important concept in the domain of the management of customer relationship (Pongpaew et al., 2017). It is well-documented that customer interaction can lead to some favorable consequences such as customer brand identification, commitment, and brand community bonding (Luo et al., 2016; Casaló et al., 2017; Heinonen et al., 2018). However, to the best of our knowledge, previous studies have yet to examine the important link between customer interaction and inspiration. Moreover, few studies examined customer interaction from the perspective of organizational climate. We offered a conceptual framework on the canvas of this study to determine the role of customer interaction in the relationship between customer inspiration and OBC climate to remove the boundaries in the understanding of customer interaction in the context of OBC.

Based on the earlier discussion, this study attempts to examine: (1) How customer inspiration is determined? (2) What are the related impacts of OBC climate on customer inspiration? (3) What are the likely associations of customer interaction with the OBC climate and customer inspiration? and (4) What is the mediating role of customer interaction between the OBC climate and customer inspiration? Thus, the objective of this study is, first, to identify and analyze the antecedents of customer inspiration in OBCs. Second, this study examines the mediating role of customer interaction between the OBC climate and customer inspiration.

To achieve the objectives of this study, we proposed a conceptual framework to test whether the OBC climate (i.e., supportive climate and controlled climate) is a determinant factor of customer interaction and customer inspiration. This study attempts to unravel the integrated mechanism of customer interaction that facilitates customer inspiration. This study bridges the research gaps in the extant literature and provides roadmaps to marketers and policymakers to utilize OBCs as a tool to strengthen the customer–brand relationship.

The remainder of this study comprises of the following sections: (1) introduction section presents the conceptual framework and hypotheses, (2) a research method to testify the proposed hypotheses, (3) empirical analysis of the gathered data and results, and (4) discussion with theoretical and practical implications followed by limitations and directions for further research.

## Literature Review

### Online Brand Community Climate

Online brand community is defined as “a specialized, non-geographically bound community based on the social relationship among admirers of a brand” in cyberspace (Muniz and O’Guinn, 2001). OBCs may include web forums, interactive websites (Hollebeek et al., 2017), and those established on social media (Habibi et al., 2014). Because of the advantages of OBCs on social media, such as transparent social context, flat structure, potentially large-scale content, and story-telling (Habibi et al., 2014), social media-based OBCs have recently gained an extensive attention from marketing scholars and practitioners (Carlson et al., 2019; Fernandes and Castro, 2020). OBCs in this study broadly refer to those online communities, which are established at different social media platforms.

In recent years, customers increasingly rely on OBCs to connect, share, and engage online with other customers and brands (Yuan et al., 2020). Notably, the number of Internet users globally was more than 4.8 billion in 2020 (Statista, 2021a). The spending on Internet advertising reached a record of 298 billion dollars globally in 2020 (Statista, 2021b). Thus, brands have started to increasingly invest in OBCs to initiate and strengthen relationships with their customers (Fernandes and Castro, 2020).

The concept of climate is defined in the field of organizational behavior as a set of measurable properties of the (work) environment, which are perceived directly or indirectly by the people who live and work in the same environment and who get influenced for their motivation and behavior (Schneider et al., 2017). This study considers OBC climate in terms of how each community member perceives and interprets the environment of the brand community. The OBC climate is also assumed to influence the attitudes and behaviors of community members. From an organizational perspective, OBCs resemble informal organizations, which are “the aggregate of the personal contacts and interactions and the associated groupings of people” (Gulati and Puranam, 2009; Huning et al., 2015; Liu et al., 2019). Scholars identify certain characteristics of OBCs, such as communication between individuals and organizations, willingness to contribute, and accomplishment of a common purpose (Muniz and O’Guinn, 2001).

Online brand communities are composed of consumers in the online environment who possess a social identification with others and share their interest in a particular brand to improve the brand image and to promote the relationship among consumers (Black and Veloutsou, 2017; Fernandes and Castro, 2020). OBCs are based on the shared consciousness of various kinds of rituals, traditions, and moral obligations to society (Black and Veloutsou, 2017). In particular, OBCs play a significant role in stimulating the emotions, such as joy, love, and positive surprise, of consumers, which in turn drive the intention of consumers to co-create the value in OBCs (Cheung et al., 2020b). Because of the commonalities between OBCs and informal organizations, this study also sheds light on the notion of organizational climate in the context of OBCs.

Drawing on the existing studies (Luthans et al., 1998; Shih et al., 2014; Zhang et al., 2021), this study considers supportive and controlled climates as a representative of the perceptions of a member of the OBC environment. A supportive climate is regarded as the level of support its members perceive or receive from the OBCs. For example, members may feel a supportive community climate when members fulfill their needs for esteem, approval, and affiliation in the community. When members get help and replies from other community members, they also feel the supportive climate of the OBCs. A supportive climate is beneficial for organizations to encourage participation in, and engagement with the organization (Luthans et al., 1998; Casaló et al., 2017; Carlson et al., 2019). In contrast, a controlled OBC climate is the one in which members perceive obstacles and impediments from the community (Bacharach and Bamberger, 2007; Shih et al., 2014). For instance, an OBC with controlled climate may influence its members to develop self-control based on the values and rules set for behaving in the OBCs, for example, to control posting of content related to advertisements and swear words. When the OBC restricts communication and collaboration or strengthens control and supervision, its members may experience this as a controlled OBC climate.

### Customer Interaction

Customer interaction refers to the extent to which customers communicate and interact with each other in OBCs (Casaló et al., 2017). Customers participate in online platforms to interact with other like-minded peers and brands to obtain value, such as entertaining value, trendiness information, and product reviews, which in turn drive the relationship among consumers (Cheung et al., 2021a). In particular, customers share, enjoy, and express themselves in the OBCs (Coelho et al., 2019), which in turn foster community relationships (Wang et al., 2013; Luo et al., 2016) and, thereby, encourage information sharing among members of the online community (Cheung et al., 2020a).

Customer interaction fuels information interaction. It is noted that information interaction refers to interfaces that are related to a product, brand, and marketing information (Pai and Tsai, 2016; Busalim et al., 2019). OBCs serve as a useful platform to seek and obtain information by allowing information exchange among customers (Pai and Tsai, 2016). The community members in OBCs share their experiences of meaningful brand consumption regarding the product usage, brand knowledge, technology, market information, and other relevant consumption-related perspectives. The experience of meaningful brand consumption means that the customers share their personal understanding of the brand through their unique experiences of consumption, which help them feel connected, important, and understood by other customers (Stach, 2019). Thus, OBCs are online platforms where information is distributed from very diverse sources (Martínez-López et al., 2017).

As a socially networked community, OBC operations are based on a structured set of social relationships among the admirers of a brand to influence the expectations and perceptions of consumers of the brand (Wang et al., 2013; Hsieh and Wang, 2016). From this, social interaction is an integral part of customer interaction in OBCs. Community members disclose personal opinions or firsthand experiences through social interaction. By doing so, they provide and receive emotional assistance and social networking support from other forum members. Consequently, this self-disclosure fosters trust and closeness among community members (Brodie et al., 2013; Coelho et al., 2019). In a recent study, it is found that brands encourage consumer–consumer interaction on OBCs to share their knowledge with each other (Cheung et al., 2021b). Thus, in this study, social interaction corresponds to interpersonal interactions among community members who are irrelevant to the brand product but are essential for the establishment and development of social relationships among the OBC members for favorable consumption behaviors (Wang et al., 2013; Luo et al., 2016).

### Relationship Between OBC Climate and Customer Interaction

Organizational climate has been the focus of previous research explaining the behavior of an individual within an organization, such as their attitudes, commitment to, and involvement in work (Hsieh and Wang, 2016; Nedkovski et al., 2017). Willingness of a member to be involved in and to socialize with others largely depends on the climate they perceive from the online platforms (Zafar et al., 2020). For example, organizational climate is a key to eliciting organizational citizenship behaviors (Marinova et al., 2019). Social climate is also positively related to social interaction, which affects different types of values of the members, such as social value, information value, and hedonic value (Sun et al., 2016). More precisely, the more positive the individuals perceive an organizational climate, the stronger their involvement will become in the organization (Zhang and Liu, 2010; Shih et al., 2014).

There is a plethora of literature that supports the strong impact of supportive organizational climate on attitudes and behaviors of individuals (Luthans et al., 1998; Hsieh and Wang, 2016), and this practice is carried over to the evaluation and attitudes of customers toward participation in OBCs (Pai and Tsai, 2016). In OBCs, a supportive climate is considered as a key component driving the interactional behaviors of members.

Situational factors play an instrumental role in shaping the interactional behaviors of individuals. A supportive climate thus is one of such situational factors in OBCs that may impact the emotions, feelings, and interaction behaviors of individuals. For instance, a climate of support generally helps members to develop a sense of brand identification and community identification, which in turn encourages members to engage with the community, either by participating in community activities or exchanging information openly and freely with each other (Tsai and Pai, 2012; Demiray and Burnaz, 2019; Kaur et al., 2020). A feeling of support thus evokes positive reactions, such as interacting more with others and co-create value in OBCs (Pai and Tsai, 2016; Matute et al., 2019; Cheung et al., 2021a). When communities are supportive and look out for concerns of members, customers sense a feeling of obligation toward the OBC and exhibit favorable engagement in online interactions (Molinillo et al., 2020).

Based on the above, we proposed the following hypotheses.

**Hypothesis H_1a_**: A supportive climate in an OBC exerts its significant and positive impact on the information interaction of a customer.

**Hypothesis H_1b_**: A supportive climate in an OBC exerts its significant and positive impact on the social interaction of a customer.

In OBCs, a controlled climate is the result of rules and regulations that prohibit members to post inappropriate content, such as swear words, advertisements, and irrelevant information (Bacharach and Bamberger, 2007; Cooper et al., 2019; Zhang et al., 2021). When OBCs place restrictions within the community, it might influence conversations and actions of community members. However, the restrictions in the controlled environment might prohibit the sharing of inappropriate content, such as violence, to respect the ethical concerns of the community members. From this, it is discerned that the controlled environments create a positive climate in the community (Cooper et al., 2019). Therefore, the majority of the community members would have a strong willingness to interact in the community in the controlled environment as well. Based on the above, we proposed the following hypotheses.

**Hypothesis H_1c_**: A controlled climate in an OBC exerts its significant and positive impact on the information interaction of a customer.

**Hypothesis H_1d_**: A controlled climate in an OBC exerts its significant and positive impact on the social interaction of a customer.

### Customer Inspiration

Inspiration is referred to as a “motivational state that compels individuals to bring ideas into fruition” (Oleynick et al., 2014, p. 1). The concept of inspiration was created and developed by Thrash and Elliot (2003). Inspiration can be analyzed as a psychological construct, comprised of three components, namely, transcendence, evocation, and motivation (Thrash and Elliot, 2003, 2004). In the consumption context, customer inspiration is noted as a motivational state that compels customers to pursue consumption-related goals after prompted by a marketing effort (Böttger et al., 2017, p. 116). Customer inspiration can be viewed as the transmission process that links the stimulus (e.g., brand experiences) and inspiration-related activities (e.g., purchase behavior) (Kwon and Boger, 2021).

From the perspective of psychology, customer inspiration is a two-step process, namely, the inspired-by state, i.e., customer inspiration by external stimuli, and the inspired-to state, i.e., customer inspiration to behave (Thrash and Elliot, 2004; Böttger et al., 2017; Kwon and Boger, 2021). The inspired-by state is an activation state that exists when a customer receives new information about the brand product, which, somehow, stimulates their imagination regarding the brand product. The inspired-by state can also be recognized as customer inspiration by external stimuli because it is the inspiration state of customers, which is influenced by an external stimulus when customers are receptive to new ideas, such as planning to purchase a product (Böttger et al., 2017). In this state, customers experience emotional elicitation that might result in their attitudinal and emotional shift toward the brand product. The inspired-to state is a goal-striving state, which stimulates the intentional purchase decisions and the consumption-related behaviors of customers (Böttger et al., 2017). The inspired-to state can be recognized as the inspiration-to behave state of a customer because it is an intrinsic pursuit of a consumption-related goal with motivation to realize the new idea (e.g., by purchasing and using a product) rather than to extend or replicate it.

Online brand communities are important platforms for customer inspiration. In the past two decades, OBCs were considered as the outlets for the quick creation and distribution of marketing information. Such marketing information is a rich source of inspiration as it yields new marketing ideas (Winterich et al., 2019). Thus, customer communications and information exchanges in OBCs have the potential to evoke customer inspiration by revealing new and better possibilities of the brand products. After communication with OBC members, customers conceive a new idea that might have not been recognized before to buy the brand product/service.

### Relationship Between Customer Interaction and Customer Inspiration (Inspired-by)

From the perspective of the social interaction theory, interaction is conceptualized as a motivational concept because it involves the ways individuals are mobilized and stimulated in interpersonal encounters (Heinonen et al., 2018). It is noted that customer interaction in OBCs significantly influences the behaviors of the customers in terms of innovation and adoption of a new technology (Shriver et al., 2013). Studies show that interactions between consumer–consumer and customer–brand have a considerable effect on driving the cognitive, emotional, and behavioral dimensions of customer-brand engagement (Cheung et al., 2021b).

The interaction of customers with each other in OBCs develops two types of relationships, namely, functional and emotional (Fernandes and Moreira, 2019). Scholars note that online interaction brings functional, cognitive, and affective benefits to customers (Hollebeek et al., 2014; Dessart et al., 2015; Heinonen et al., 2018).

Extant literature on inspiration suggests that inspiration is triggered by an external stimulus (Kwon and Boger, 2021). In the context of OBCs, customer interaction serves as an inspiration-evoking source. It is because customers might experience moments of inspiration after interacting with others in OBCs. Thus, customer interactions (e.g., information and social interaction) in OBCs lead to stimulating customer inspiration, in particular, the inspired-by state of customers, which represents that imaginations and mental horizons of customers about a brand product are stimulated as a consequence of customer interactions.

The fact that information interaction in OBCs leads to perceived cognitive benefits of customers is well-documented in previous studies (Nambisan and Baron, 2009; Wang et al., 2013). From the perspective of the online environment, an inspirational content inspires customers who are connected to online social media (Izogo et al., 2020). Scholars document that product–content interactions bring cognitive and learning benefits to customers, such as knowledge on product usage, brand information, technology, and market-related information (Nambisan and Baron, 2009; Luo et al., 2016). Customers gain new insights into brands with a high degree of information interaction that might facilitate the communication, sharing, and transfer of knowledge among customers of OBCs (Pai and Tsai, 2016).

The versatility and interactions of customers in OBCs generally fuel the process of the generation of a new idea to develop products (Wu and Fang, 2010). This phenomenon of information exchange and interaction helps customers with quick solutions for certain technical problems alongside gaining new knowledge about the brand product/service. Therefore, information interaction, which delivers valuable and helpful messages to customers, has the potential to inspire customers by broadening their horizons about the brand product/service.

Based on the above, the following hypothesis is proposed.

**Hypothesis H_**2a**_**: Information interaction in an OBC exerts its significant and positive impact on the inspiration of a customer (i.e., inspired-by).

Social interaction in OBCs may also drive customer inspiration (i.e., inspired-by). Frequent social interactions help customers build and strengthen social ties and a sense of belongingness with other customers in OBCs (Zaglia, 2013; Yoshida et al., 2018). From a psychological perspective, positive consumer experiences in OBCs lead to customer satisfaction and identification with the community, which increase community cohesiveness (Lin et al., 2019). Likewise, when customers develop harmonious community relationships, they more likely share their personal opinions and experiences with other customers in OBCs (Luo et al., 2016). Moreover, brands act as relationship builders in OBCs and promote customer interaction (Veloutsou and Ruiz Mafe, 2020). It is noted that the committed interactions of customers in OBCs strengthen the notions of brand commitment, loyalty, and the valuable experience of customers (Zhang et al., 2020). The customers reinforce their sense of band identity as their social interaction unfurls in OBCs. This promotes the passion of customers for the brand and influences their attitude and commitment toward the consumption of brand products (Wang et al., 2013). Based on the above, we proposed the following hypothesis.

**Hypothesis H_2b_**: Social interaction in an OBC exerts its significant and positive impact on the inspiration of a customer (i.e., inspired-by).

### Relationship Between Inspired-by and Inspired-to States of Customer Inspiration

The transmission model of inspiration presents that the inspired-by and inspired-to states of inspiration of customers are two components that are causally linked where inspired-by triggers the inspired-to component (Thrash and Elliot, 2004; Böttger et al., 2017; Kwon and Boger, 2021). This causal relationship is elaborated in the environments of customer purchasing; customers are inspired by marketing stimuli, and they are subsequently inspired to act (Böttger et al., 2017).

From the perspective of consumer psychology, customers thus make a transition from the state of being inspired by a new idea to the state of being inspired to implement this idea. Generally, customers will have a greater motivation to act based on new information that raises the awareness of customers regarding new or better possibilities of a brand product/service (Winterich et al., 2019). Inspiration motivates customers to act according to their filtered idea through inspirational messages (Liang et al., 2016). Therefore, we presented the following hypothesis:

**Hypothesis H_3_**: The inspired-by state of customers exerts its significant and positive impact on their inspired-to state.

Based on the above-discussed associations of customer inspiration with OBC climate and customer inspiration, we proposed the following hypotheses.

**Hypothesis H_4a_**: Information interaction significantly mediates the relationship between supportive climate of an OBC and inspiration of customers (i.e., inspired-by).

**Hypothesis H_4b_**: Social interaction significantly mediates the relationship between supportive climate of an OBC and inspiration of customers (i.e., inspired-by).

**Hypothesis H_4c_**: Information interaction significantly mediates the relationship between controlled climate of an OBC and inspiration of customers (i.e., inspired-by).

**Hypothesis H_4d_**: Social interaction significantly mediates the relationship between controlled climate of an OBC and inspiration of customers (i.e., inspired-by).

**FIGURE 1:**
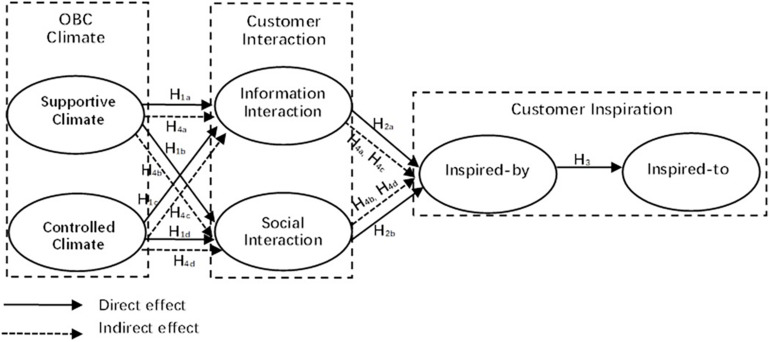
The conceptual model.

Based on the discussed theoretical underpinnings, we presented a conceptual framework of the study (Figure 1).

## Methodology

### Survey Instrument

To test and validate the proposed hypotheses, a survey questionnaire was developed in this study to gather data. The survey questionnaire was divided into two parts. The first part measured items of the proposed constructs of the study, i.e., OBC climate (including supportive and controlled climate), customer interaction (including information and social interaction), and customer inspiration (including inspired-by and inspired-to states). The second part contained items of demographic information of the study respondents.

The survey questionnaire items were adopted from the previous research, i.e., scale items for inspired-by and inspired-to in customer inspiration were adopted from Böttger et al. (2017), for example, “My imagination was stimulated” for “inspired-by” and “I was inspired to buy something” for “inspired-to.” Scale items for supportive climate were adapted from Rogg et al. (2001), such as “Members in the brand community are treated equally and with respect.” Scale items for information interaction and social interaction were adapted from Luo et al. (2016) and Wang et al. (2013), such as “I share my brand and product knowledge with others in the community” for information interaction and “I always participate in two-way communications for sharing experiences and feelings” for “social interaction.”

We developed new scale measures for the construct of “controlled climate” due to its novelty. For this, we followed the guidelines of Gilbert (1979). A total of four measurement items for “controlled climate” were filtered from the extant literature (Bacharach and Bamberger, 2007; Martínez-López et al., 2017). For example, “you are not allowed to misbehave in the company.” A total of 12 marketing professors were invited to comment on the gathered measurement items. The panel of experts suggested to delete one scale item and helped to revise and improve the remaining three finalized scale items of controlled climate. All scale items were adjusted according to the context of the study. For instance, one item which is described as “there are certain rules to follow in the company” and was adjusted as “there are certain rules to follow in this community” as this study is conducted in the context of mobile phone OBCs.

All scale items were measured on a 7-point Likert scale, where 1 presented “strongly disagree” and 7 presented “strongly agree.” All of the scale items were originally developed in English. We invited two postdoctoral fellows, who were native Chinese speakers and fluent in English language, to translate the scale items into Chinese using the blind translation-back translation method (Soriano and Foxall, 2002).

To determine the competency of the developed questionnaire, we conducted a pilot study in Shenzhen. A total of 32 volunteer individuals were recruited to answer the survey questionnaire by posting information of the study in different groups of WeChat,^1^ which is one of the leading social media in China. Findings show an acceptable level of Cronbach’s alpha (α) values for the study constructs, i.e., >0.70 (Anderson and Gerbing, 1988). However, the questionnaire items were little adjusted to improve the general understanding of the questionnaire based on the thoroughly reviewed feedback of respondents. For instance, several words were replaced by terms more suitable for OBC contexts, such as “posts” and “moderators.”

### Data Collection

We considered the OBCs of mobile phones as the study context due to their prevalence in China. An online survey was conducted to gather data from the Chinese respondents who were members of OBCs for mobile phones with an interest in online purchase of brand products. The study respondents with the age of at least 18 years were considered to ensure the consent requirement of respondents (Majeed et al., 2020). The data were collected at Wenjuan Xing,^2^ which is one of the leading platforms to conduct online surveys, between May 29, 2020 and June 14, 2020. Wenjuan Xing administered the survey by adopting a random sampling method to gather data from its more than three million samples with diverse demographic backgrounds from a variety of Chinese cities. This platform for online data collection adopts a multichannel distribution of questionnaires and randomly invites users with integration of WeChat groups and red envelop lottery as an incentive to fill the questionnaires. Thus, the gathered data offer a greater representation of the relevant population in this study.

To select the appropriate respondents, we added two screening questions before the questionnaire items, namely, (1) What is the name of an OBC you participate to interact with other people to purchase mobile phones? (2) Do you frequently visit your preferred OBC? Only respondents who answered “yes” to the question regarding frequent visitation in their mentioned OBC were considered for participation in the study. We collected a total of 524 responses during the data collection process. After excluding the negative answers to the screening questions and incomplete responses, a total of 504 responses were retained for the analysis of final data.

### Demographic Details of Samples

The demographic profiles of respondents (Table 1) showed that 211 respondents (41.86%) were males and 293 respondents (58.14%) were females. The majority of respondents aged between 26 and 30 years (i.e., 141, 27.98%), held an undergraduate degree (i.e., 380, 75.4%), had monthly income between ¥ 6,001 and ¥ 8,000 (i.e., 141, 20.04%), and held the membership of OBC approximately 2–3 years ago (i.e., 147, 29.17%).

**TABLE 1:** Respondents’ demographic detail.

*N* = 504	Frequency	Percentage	Cumulative percentage
**Gender**			
Male	211	41.86%	41.86%
Female	293	58.14%	100%
**Age**			
Below 20	39	7.74%	7.74%
21–25	124	24.6%	32.34%
26–30	141	27.98%	60.32%
31–35	140	27.78%	88.1%
36–40	40	7.94%	96.04%
41–45	12	2.38%	98.42%
46–50	5	0.99%	99.40%
51 and above	3	0.60%	100%
**Education**			
Below High school	19	3.77%	3.77%
Junior college	45	8.93%	12.7%
Undergraduate	380	75.40%	88.1%
Master	59	11.71%	99.80%
Doctor	1	0.20%	100%
**Income (RMB)**			
Less than 2000	63	12.50%	12.50%
2,001–4,000	55	10.91%	23.41%
4,001–6,000	75	14.88%	38.29%
6,001–8,000	101	20.04%	58.33%
8,001–10,000	99	19.64%	77.98%
10,001–12,000	54	10.71%	88.69%
Above 12,000	57	11.31%	100%
**OBC membership**			
Below 1 year	35	6.94%	6.94%
1–2 years	138	27.38%	34.33%
2–3 years	147	29.17%	63.49%
3–4 years	101	20.03%	83.53%
4–5 years	45	8.93%	92.46%
More than 5 years	38	7.54%	100%

### Control Variables

In order to evaluate the proposed research model, we controlled four relevant descriptive statistic variables, namely, gender, age, education, and membership tenure, which may impact customer inspiration in OBCs. It is because individual characteristics of people may play a significant role in influencing the frequency and intensity of the inspiration experience (Thrash and Elliot, 2004). People with different educational backgrounds may exhibit varying levels of cognitive ability, which could impact their inspirational experience (Thrash and Elliot, 2004; Kwon and Boger, 2021). Membership tenure of OBCs may create more chances for the members of OBCs to communicate and interact with each other, which may result in close interpersonal relationships and may impact the interaction intention to a certain extent (Zhou et al., 2012).

## Results

### Common Method Bias

Respondents anonymously answered the questionnaire in this study. This approach reduced the psychological resistance and common method bias of respondents (Podsakoff et al., 2003). Reverse statements were included in the questionnaire to keep the attention of respondents in reading the statements. Further, Harman’s one-factor was used to analyze the common method variance (CMV) (Podsakoff et al., 2003). The results showed that the first principal component explained only 32.03% of the variance indicating traces of common method bias in this study, which is acceptable without any potential threat according to the guidelines of Podsakoff et al. (2003). Moreover, CMV single model fit was poor [normal chi-square/degrees offreedom (CMIN/DF) = 7.249; comparative fit index (CFI) = 0.715; Tukey–Lewis index (TLI) = 0.688; incremental fit index (IFI) = 0.716; relative fit index (RFI) = 0.655; normed fit index (NFI) = 0.685; root mean square residual (RMR) = 0.121; and root mean square error of approximation (RMSEA) = 0.111] (Hulland et al., 2018). This shows that the results of this study were not confounded by common method bias, and the model provides a good fit for the data.

### Evaluation of Measurement Model

This study analyzes the gathered data using the AMOS 22 software. According to the two-step approach of structural equation modeling (Anderson and Gerbing, 1988), factor loadings great than 0.50 were considered significant. Findings show that three items for “supportive climate” (including the brand community supports innovative post, the brand community is criticism-taking, and the brand community takes action on good ideas provided by its members) and one item for “controlled climate” (including negative posts related to the brand product will be reviewed in the brand community X) were insignificant. After deleting insignificant items, standardized factor loadings of remaining items are presented in Table 2.

**TABLE 2:** Measure items, the reliability, and convergent validity.

Items	Standardized factor loadings	α	CR	AVE
Supportive climate (SC)				
Members in the brand community are treated equally and with respect.	0.732			
Members in the brand community trust each other.	0.784			
Members in the brand community cooperate to get the problem solved.	0.694	0.846	0.854	0.539
Members in the brand community have a good relationship with others.	0.718			
The brand community encourages communication and collaboration.	0.738			
Controlled climate (CC)				
If any member posts advertisements, the forum moderator will give warnings, and delete the post.	0.717			
If any member misbehaves (e.g., insulting other members), the forum moderator will give warnings or forbid him/her to post, etc.	0.793	0.766	0.767	0.524
There are certain rules to follow in this community.	0.656			
Information interaction (II)				
My community interaction contained large amount of information about the brand and product usage (e.g., product feature, functions, and updates).	0.757			
My community interaction contained large amount of information about product technology (e.g., standards) and product market (e.g., competing products and pricing)	0.733	0.797	0.795	0.565
I share my brand and product knowledge with others in the community.	0.765			
Social interaction (SI)				
I always post new threads in the community and will get response quickly from others.	0.796	0.821	0.820	0.535
I always actively take part in community discussions and have close and intensive interactions with other members of the online brand community.	0.728
I always participate in two-way communications for sharing experiences and feeling etc.	0.728
I take part in some recreational posts.	0.668
Inspired-by	
My imagination was stimulated.	0.737			
I was intrigued by a new idea.	0.773			
I unexpectedly and spontaneously got new ideas.	0.730	0.863	0.862	0.557
My horizon was broadened.	0.755			
I discovered something new.	0.735			
Inspired-to				
I was inspired to buy something.	0.745			
I felt a desire to buy something.	0.794			
My interest to buy something was increased.	0.753	0.866	0.867	0.568
I was motivated to buy something.	0.784			
I felt an urge to buy something.	0.689			

*α = Cronbach’s alpha; CR = composite reliability; AVE = average variance extracted.*

Findings of the measurement model show acceptable fit, i.e., CMIN/DF = 1.983; CFI = 0.958; TLI = 0.951; IFI = 0.958, RFI = 0.906, NFI = 0.919; RMR = 0.059; RMSEA = 0.044, and, thus, present adequate reliability and validity of data (Kumar, 2021). Cronbach’s alpha (α) values were found greater than the threshold of 0.70 (Anderson and Gerbing, 1988) indicating a good internal consistency among all the items of the constructs and the reliability of the model. Findings of composite reliability (CR) of the measurement model were greater than the recommended threshold of 0.60 (Bagozzi and Yi, 1988), showing a good internal reliability.

The discriminant validity was assessed using the average variance extracted (AVE). The results reveal the internal consistency (i.e., α, CR, and AVE) of the measurement model. Additionally, all the square roots of AVEs exceeded the coefficients between each pair of constructs (see Table 3).

**TABLE 3:** Mean, standard deviation, and correlation matrix.

Construct	SC	CC	II	SI	Inspired-by	Inspired-to
SC	0.734					
CC	0.266	0.724				
II	0.470	0.319	0.752			
SI	0.488	0.320	0.451	0.731		
Inspired-by	0.525	0.342	0.596	0.565	0.746	
Inspired-to	0.428	0.298	0.498	0.530	0.610	0.754
Mean	5.502	5.710	5.612	5.154	5.369	5.369
SD	0.853	1.006	0.952	1.070	0.941	1.013

*N = 504, SC = supportive climate; CC = controlled climate; II = information interaction; SI = social interaction; the values on the diagonal are the AVE square root of each construct.*

### Evaluation of Structural Model

In the second step, we tested the hypotheses H_1_ (i.e., H_1__a_, H_1__b_, H_1__c_, and H_1__d_), H_2_ (i.e., H_2__a_ and H_2__b_), and H_3_. Overall, the model exhibited acceptable fit (Kumar, 2021), (CMIN/DF = 1.850; CFI = 0.963; RFI = 0.912; NFI = 0.923; IFI = 0.963; RMSEA = 0.041) Hypotheses H_1__a_ and H_1__b_ regarding the significant and positive relationship between an OBC supportive climate and customer interaction were supported (information interaction: β = 0.579, *t* = 10.123, *p* < 0.001; social interaction: β = 0.553, *t* = 8.169, *p* < 0.001). Findings show that a controlled OBC climate positively influences customer interaction (information interaction: β = 0.269, *t* = 4.815, *p* < 0.001; social interaction: β = 0.207, *t* = 3.786, *p* < 0.001). Thus, the second set of hypotheses (H_1__c_ and H_1__d_), which show a positive effect of a controlled OBC climate on customer interaction, are supported.

Findings show that information interaction and social interaction have a significantly positive impact on the inspired-by component of customer inspiration (information interaction: β = 0.556, *t* = 9.802, *p* < 0.001; social interaction: β = 0.361, *t* = 7.861, *p* < 0.001). Thus, hypotheses H_2__a_ and H_2__b_ are supported. Moreover, findings support the significantly positive impact of inspired-by state on inspired-to state of customer inspiration, i.e., β = 0.931, *t* = 12.203, *p* < 0.001. Thus, hypothesis H_3_ is supported. Findings are summarized in Table 4.

**TABLE 4:** Results of hypothesized model.

Hypothesis	Path	Proposed effect	Path coefficient	*P*-value	Results
H_1__a_	SC → II	Positive	0.579	<0.001	Supported
H_1__b_	SC → SI	Positive	0.553	<0.001	Supported
H_1__c_	CC → II	Positive	0.269	<0.001	Supported
H_1__d_	CC → SI	Positive	0.207	<0.001	Supported
H_2__a_	II → inspired-by	Positive	0.556	<0.001	Supported
H_2__b_	SI → inspired-by	Positive	0.361	<0.001	Supported
H_3_	Inspired-by → inspired-to	Positive	0.931	<0.001	Supported

*SC = supportive climate; CC = controlled climate; II = information interaction; SI = social interaction.*

### Mediation Test

We used the bootstrap method (with 5,000 samples) with 90% bias-corrected confidence intervals to examine the mediating role of customer interaction (i.e., information and social interaction) between OBC climate (i.e., supportive and controlled climate) and customer inspiration (i.e., inspired-by). Findings (Table 5) show a significant standardized indirect effect of OBC supportive climate on customer inspiration (β_information interaction_ = 0.269, β_social interaction_ = 0.144) at zero confidence interval, i.e., for information interaction: confidence interval (CI)_u__pper_ = 0.351, CI_lower_ = 0.197; for social interaction: CI_u__pper_ = 0.213, CI_lower_ = 0.093, supporting hypotheses H_4__a_ and H_4__b_. The standardized direct effect of supportive climate on customer inspiration (i.e., inspired-by) was insignificant (*p* = 0.113) at zero confidence interval (β = 0.132, CI_u__pper_ = 0.264, CI_lower_ = -0.004) presenting a complete mediating impact of information interaction and social interaction on the relationship between supportive climate and customer inspiration (i.e., inspired-by).

**TABLE 5:** Mediation test.

Relationship	β	Bias-corrected 90% CI		*p* value
		Lower	Upper	
SC → II → Inspired-by	0.269	0.197	0.351	*p* < 0.001
SC → SI → Inspired-by	0.144	0.093	0.213	*p* < ‘0.001
CC → II → Inspired-by	0.066	0.038	0.108	*p* < 0.001
CC → SI → Inspired-by	0.147	0.094	0.211	*p* < 0.001
SC → Inspired-by	0.132	–0.004	0.264	*p* = 0.113
CC → Inspired-by	0.058	–0.039	0.169	*p* = 0.303

Findings present a significant standardized indirect effect of OBC controlled climate on customer inspiration (β_information interaction_ = 0.066, β_social interaction_ = 0.147) at zero confidence interval, i.e., for information interaction: CI_u__pper_ = 0.198, CI_lower_ = 0.038; for social interaction: CI_u__pper_ = 0.211, CI_lower_ = 0.094, supporting hypotheses H_4__c_ and H_4__d_. The standardized direct effect of controlled climate on customer inspiration (i.e., inspired-by) was insignificant (*p* = 0.303) at zero confidence interval (β = 0.132, CI_u__pper_ = 0.169, CI_lower_ = −0.039) presenting complete mediating impact of information interaction and social interaction on the relationship between supportive climate and customer inspiration (i.e., inspired-by). Findings are reported in Table 5.

## Discussion

This study investigates customer inspiration in OBCs. Customer inspiration is an important psychological state that a customer may experience. Findings help to validate the research of this study. First, the results and analyses show that OBC climate and customer interaction are two important antecedents of customer inspiration in terms of their direct and indirect impacts. Second, two types of OBC climate, including supportive climate and controlled climate, are significantly and positively related to customer inspiration. Third, customer interaction, including social and information interaction, exerts its mediating impact on the relationship between OBC climate (i.e., supportive and controlled climate) and customer inspiration (i.e., inspired-by). Last, customer interaction is significantly and positively related to customer inspiration.

More precisely, our findings support that there is a significant and positive relationship between supportive climate and customer interaction. Previous studies revealed that there exists a positive relationship between supportive climate and customer interaction in offline environments (Shih et al., 2014). Based on the empirical analysis, this study presents that a supportive climate in virtual spaces, such as WeChat and TikTok, also plays an important role in promoting customer interaction. In particular, the higher levels of perceived support from online communities help to increase the sense of belonging of the community members (Matute et al., 2019), which leads customers to communicate and interact more with each other.

This study highlights the impact of a controlled climate on the interactions of customers in brand communities (Bacharach and Bamberger, 2007). This is because a controlled climate such as forbidding members to advertise for their personal products helps to regulate and purify the community environment, which encourages member interaction in OBCs. There might be a possibility that a small number of members of OBCs feel restrained due to the controlled environment because they are not allowed to post certain contents such as advertisements and violent contents in the online communities. Nevertheless, the majority of the community members, in contrast, have more willingness to interact with each other in the controlled climate of OBCs.

Through interactions, customers might not only gain new information and knowledge but also develop attachment and loyalty to the brand. From the perspective of OBCs, the findings of this study provide support to the positive relationship between customer interaction and customer inspiration (i.e., inspired-by). The inspired-by state of customer inspiration also showed a significant and positive association with the inspired-to state. This shows that customers purchase a product if they feel inspired in the consumption context. These results are in line with the findings of Böttger et al. (2017).

### Theoretical Implications

This study offers several theoretical implications. First, this study puts forth a novel concept of OBC climate by unraveling the instrumental association between organizational climate and OBC. With the profound empirical analysis, this study updates existing literature boxes of organizational climate and OBC and develops important theoretical underpinnings of the field. The notion of “organizational climate” is a mature concept in the domain of organizational psychology and behavior (Schneider et al., 2017). However, this concept remained under-explored from the perspective of OBC. Drawing on the theoretical understanding of organizational climate (Coda et al., 2015; Sun et al., 2016; Zafar et al., 2020), this study shows that OBCs also involve organizational climate. This study not only extends the theoretical understanding of two streams of literature, namely, organization climate and OBC, but also equips scholars to conduct future research on OBC climate under the empirical lens of this study.

Second, this research examines customer inspiration in the context of OBCs to develop theoretical knowledge in the domain of OBCs. A plethora of studies exist that examine OBC from different perspectives, such as community tribalism, identification, and customer engagement and value co-creation (Wirtz et al., 2013; Hollebeek et al., 2014; Black and Veloutsou, 2017; Coelho et al., 2019). However, the instrumental phenomenon of customer inspiration in buying brand products in OBCs remained widely under-explored (Winterich et al., 2019; Izogo and Mpinganjira, 2020). This study highlights the importance of OBCs for customer inspiration and investigates OBCs from an organizational climate perspective. Therefore, this study contributes to brand community literature by incorporating customer inspiration and, thus, attempts to fill the theoretical gaps in the field of consumer behavior, marketing, and brand management in the online environment. Thus, this study opens the boundaries of OBC literatures by combing different research perspectives, such as psychology, and consumer behavior to interpret the notions of inspiration in psychology and climate in organizational studies.

Third, this study contributes to the organizational climate literature by incorporating the notion of controlled climate with supportive climate. Previous studies on organizational behavior mainly focused on the role of supportive climate, such as its impact on job performance of employers and organization commitment (Rogg et al., 2001; Bacharach and Bamberger, 2007; Zhang and Liu, 2010; Shih et al., 2014; Molinillo et al., 2020) and neglected the important stimulus of controlled climate in shaping consumer behavior. We examined the role of controlled climate with supporting climate in OBCs and offered shreds of evidence on the empirical grounds of this study. Although a controlled climate impacts a small number of OBC members, overall, it benefits the majority of OBC members and the OBC to regulate and purify the OBC environment. A controlled climate acts as external stimuli that affect the perceived trust and norms of reciprocity of community members and then drive them to engage with the OBCs (Zhang et al., 2021). Without proper control and regulation, there may exist risks of harassment, bullying, and other safety problems for members in OBCs. Thus, this study highlights the importance of a controlled climate in OBCs for encouraging interactions among the members of OBCs, which influence their inspiration to buy brand products.

### Managerial Implications

One of the most important goals for brands and marketers is to inspire customers to buy their products. The findings of this study uncover the importance of OBCs to achieve this goal. This study presents the woven web of OBCs, customer interaction, and customer inspiration to provide robust insights to brand managers and marketers for the promotion of brand products in the online environment.

First, this study shows the importance of OBCs to policymakers, marketers, and brand managers the coordinating mechanism between OBCs and customer interaction to stimulate customer inspiration. This study illuminates the pathways for the effective management of the brand–customer relationship. The findings of this study are in line with previous studies that encourage companies to engage OBCs to interact with customers to improve the brand–customer relationships, such as increase in the brand community identification (Kaur et al., 2020), brand attachment (Dwivedi et al., 2019) and value co-creation behaviors of customers (Sanz-Blas et al., 2019) as a strategy to promote brand–customer relationships.

Online brand communities provide a platform to brands and marketers to present new and surprising (i.e., product and marketing) ideas to customers to stimulate the inspired-by component of customer inspiration. Marketers of brands can utilize OBCs for the distribution of their advertising messages. OBC managers may inspire customers by presenting existing products in new or unexpected combinations and forms. For example, Xiaomi Company presents its products (e.g., Xiaomi phone, TV, air conditioner, and intelligent door lock) in different combinations to show its customers how to create an intelligent home system. Moreover, companies can also use new technologies, such as AR, visual search, and other online tools, to better support the creation of the visual content of the product in OBCs.

The introduction of information communication technology, the Internet, and the virtual markets has fueled the function of online marketing better than earlier. This elaborates the growing importance of OBCs to interact with customers and to inspire them to buy the products of a brand. Given the strong competition among companies to get a big slice of market share from the online business, the marketers of brands may capitalize on the changing context of virtual buying and may focus on the effective management of customer relationships at online platforms, such as OBCs.

To improve the working mechanism of OBCs, the administrators of OBCs need to create a balanced OBC climate in which members feel free to communicate and interact with each other with controlled norms. OBC administrators and brand marketers may collaborate to appropriately understand motivations and needs of customers, for example, the search for new information, product, emotional support, a solution to specific problems, establish a relationship, etc., with intentions to promote the products of a brand from the perspective of addressing the needs of customers. This will help to develop supportive climate in OBCs with controlled climate norms, such as the monitoring and feedback from OBC administrators and brand marketers. By doing so, OBC administrators may offer incentives to stimulate supportive behaviors of customers toward other customers. For example, in Huawei Pollen community, a community member is rewarded with some forum gold if he/she helps to solve the problem of other members of the community. The measures like this may positively influence the perceptions of users of OBCs and may pump-up their active participation in OBCs.

At the same time, a controlled climate is important in OBCs due to the risks of unethical practices from the members and companies, for example, posting racial contents or to judge the integrity of community members, which may arise without proper group norms and controls (Liu et al., 2019). A controlled climate in OBCs may help to develop a harmonious community in which members will feel safe and free to interact with each other. The administrators of OBC should try to create the conditions most conducive to customer interaction. For instance, TikTok also lists several community guidelines that prohibit its members to post certain content at the platform, such as violent extremism, illegal activities, hateful behavior, adult nudity, and sexual activities. These types of restrictive community rules help to purify the overall climate of online social media platforms for better communication. However, OBC administrators need to keep a balance between supportive climate and controlled climate to improve the overall environment of OBCs.

Third, marketers need to focus on customer interaction when managing OBCs. Managing the online customer interactions is of paramount importance for brand marketing managers. It is because online customer communications and interactions are effective in promoting customer purchase behaviors (Ghahtarani et al., 2020). Thus, the design of OBC interface must be interactive and supportive to users to easily engage them in conversations with others by focusing on problem-solving, providing feedback, or fostering discussions on relevant topics. Brands may take proactive roles to engage community members in discussions and may offer their support and volunteer service to customers. This will encourage information interaction of customers alongside social interaction with brands. Customer–brand interaction may develop the sense of belonging of customers with brands which may, ultimately, improve the customer–brand relationship.

### Limitations and Future Research Directions

The present study has several limitations, which provide directions for the future research. First, the conceptualization of OBC climate is still at its infant stage. There is a need for more research to be carried out to explore the impact of OBC climate. Second, this study examines the role of OBCs as one of the marketing platforms to stimulate customer inspiration. Future research may focus on other antecedents of customer inspiration to analyze consumer behavior and customer–brand relationship by following the guidelines of this study, e.g., OBC value congruity and brand community psychological ownership, to extend the scope of this study. Third, this study is conducted in China on the Chinese customers who were having membership of OBCs. National culture may influence the behaviors of customers. Future research can be conducted in other geographical settings to gain insights from cross-culture variations.

## Data Availability Statement

The datasets generated for this study are available on request to the corresponding author.

## Ethics Statement

Ethical review and approval was not required for the study on human participants in accordance with the local legislation and institutional requirements. However, informed consent was obtained from all subjects involved in the study.

## Author Contributions

YC and ZZ contributed to the conception and design of the study. YC performed the statistical analysis and wrote the first draft of the manuscript. ZZ and SM revised the manuscript. All authors have read and agreed to the published version of the manuscript.

## Conflict of Interest

The authors declare that the research was conducted in the absence of any commercial or financial relationships that could be construed as a potential conflict of interest.

## Publisher’s Note

All claims expressed in this article are solely those of the authors and do not necessarily represent those of their affiliated organizations, or those of the publisher, the editors and the reviewers. Any product that may be evaluated in this article, or claim that may be made by its manufacturer, is not guaranteed or endorsed by the publisher.

## Footnotes

1
https://www.wechat.com/

2
http://www.wjx.com
